# Optimal allogeneic islet dose for transplantation in insulin-dependent diabetic *Macaca fascicularis* monkeys

**DOI:** 10.1038/s41598-021-88166-y

**Published:** 2021-04-21

**Authors:** Geun Soo Kim, Chan Woo Cho, Jong Hyun Lee, Du Yeon Shin, Han Sin Lee, Kyo Won Lee, Yeongbeen Kwon, Jae Sung Kim, Heung-Mo Yang, Sung Joo Kim, Jae Berm Park

**Affiliations:** 1 Department of Health Sciences and Technology, Samsung Advanced Institute for Health Sciences and Technology, Graduate School, Sungkyunkwan University, Seoul, Republic of Korea; 2 Stem Cell and Regenerative Medicine Institute, Samsung Medical Center, Seoul, Republic of Korea; 3 Transplantation Research Center, Samsung Medical Center, Seoul, Republic of Korea; 4 Department of Surgery, Yeungnam University College of Medicine, Daegu, Republic of Korea; 5 Department of Surgery, Samsung Medical Center, Sungkyunkwan University School of Medicine, 81 Irwon-ro, Gangnam-gu, Seoul, 06351 Republic of Korea; 6 Division of Endocrinology and Metabolism, Department of Medicine, Samsung Medical Center, Sungkyunkwan University School of Medicine, Seoul, Republic of Korea; 7 Department of Medicine, Sungkyunkwan University School of Medicine, Gyeonggi, Republic of Korea; 8 GenNBio Inc, Gyeonggi, Republic of Korea

**Keywords:** Immunology, Endocrinology

## Abstract

Many groups are working to improve the results of clinical allogeneic islet transplantation in a primate model. However, few studies have focused on the optimal islet dose for achieving normal glycemia without exogenous insulin after transplantation in primate models or on the relationship between rejection and islet amyloid polypeptide (IAPP) expression. We evaluated the dose (10,000, 20,000, and > 25,000 islet equivalents (IEQ)/kg) needed to achieve normal glycemia without exogenous insulin after transplantation using eleven cynomolgus monkeys, and we analyzed the characteristics exhibited in the islets after transplantation. 10,000 IEQ/kg (N = 2) failed to control blood glucose level, despite injection with the highest dose of exogenous insulin, and 20,000 IEQ/kg group (N = 5) achieved unstable control, with a high insulin requirement. However, 25,000 IEQ/kg (N = 4) achieved normal glycemia without exogenous insulin and maintained it for more than 60 days. Immunohistochemistry results from staining islets found in liver biopsies indicated that as the number of transplanted islets decreased, the amount of IAPP accumulation within the islets increased, which accelerated CD3^+^ T cell infiltration. In conclusion, the optimal transplantation dose for achieving a normal glycemia without exogenous insulin in our cynomolgus monkey model was > 25,000 IEQ/kg, and the accumulation of IAPP early after transplantation, which depends on the transplanted islet dose, can be considered one factor in rejection.

## Introduction

Islet transplantation is a feasible therapeutic option for patients with type 1 diabetes mellitus (T1DM) that can offer optimal glycemic control and prevent severe hypoglycemia. The Edmonton protocol demonstrated that transplanting more than 10,000 islet equivalents per kilogram of body weight (IEQ/kg) cured T1DM patients, although all patients required islets from two deceased-donor pancreases^1^. Clinical islet transplantation faces several remaining challenges, including a shortage of human donors. The current technique for islet isolation cannot obtain the maximum number of islets available from each pancreas donor^2,3^. After intra-portal infusion, a considerable number of islets is lost to an instant blood-mediated inflammatory reaction^4–6^. In addition, immunosuppressive drugs applied after islet transplantation can be diabetogenic^7,8^. To overcome those clinical obstacles, researchers have used nonhuman primates (NHPs) as surrogates for humans because of their evolutionary proximity, immune systems similar that of humans, and larger body sizes and longer lifespans, compared with other experimental animals^9–13^.

When studying allogeneic islet transplantation using NHPs, the number of islets required for insulin independence is important information for which limited evidence is available. Furthermore, the environment of transplanted islets and how it changes according to the infused islet dose should also be clarified. Several studies of allogeneic islet transplantation in primates reported various results, depending on the choice of immunosuppressants. Studies using JAK3 inhibitors (3900–12,500 IEQ/kg)^14^ or anti-CD40 (9361–32,387 IEQ/kg)^15,16^ immunosuppression after allogeneic islet transplantation achieved normal blood glucose levels with or without a significant reduction in the amount of exogenous insulin required. In the results of a study that transplanted 20,000 IEQ/kg allogeneic islets, the group that received co-transplantation with bone marrow–derived spheroids (to improve transplant prognosis), achieved normal glucose levels without exogenous insulin and maintained it for a period. However, the control group that received only islets did not achieve a normal blood glucose level without exogenous insulin^17^. In our previous study, the surface manipulation of islets to improve the results of allogeneic islet transplantation (9000–20,000 IEQ/kg) also did not produce insulin-independent blood glucose level normalization in the control groups (10,000 and 20,000 IEQ/kg)^18^. However, a study using an immunosuppressive protocol similar to the one we used in this study reported achieving a normal blood glucose level without exogenous insulin in the group that received 2–30,000 IEQ/kg^19^.

Meanwhile, various results have been made in determining the cause of T1DM, and islet amyloid polypeptide (IAPP) is one possibility. IAPP is well known for its role in type 2 diabetes. It is produced with insulin in the beta cells through the endoplasmic reticulum and Golgi and is an oligomer that exhibits intracellular toxicity, ultimately increasing apoptosis and destroying beta cells^20–24^. For this reason, the opinion that IAPP is the main cause of type 2 diabetes is highly convincing. IAPP has also received attention as a cause of beta-cell dysfunction in T1DM^25–27^, and some studies have reported a relationship between T1DM and IAPP^28–31^. However, within our knowledge, no studies have examined the correlation between IAPP and the dose of transplanted islets.

Our aim in the present study was to establish the optimal allogeneic islet transplantation dose to normalize the blood glucose level without requiring exogenous insulin in cynomolgus monkeys and to explain the correlation between the dose of transplanted islets and the IAPP expressed in those islets.

## Results

### Transplant-recipient monkeys

**Figure 1:**
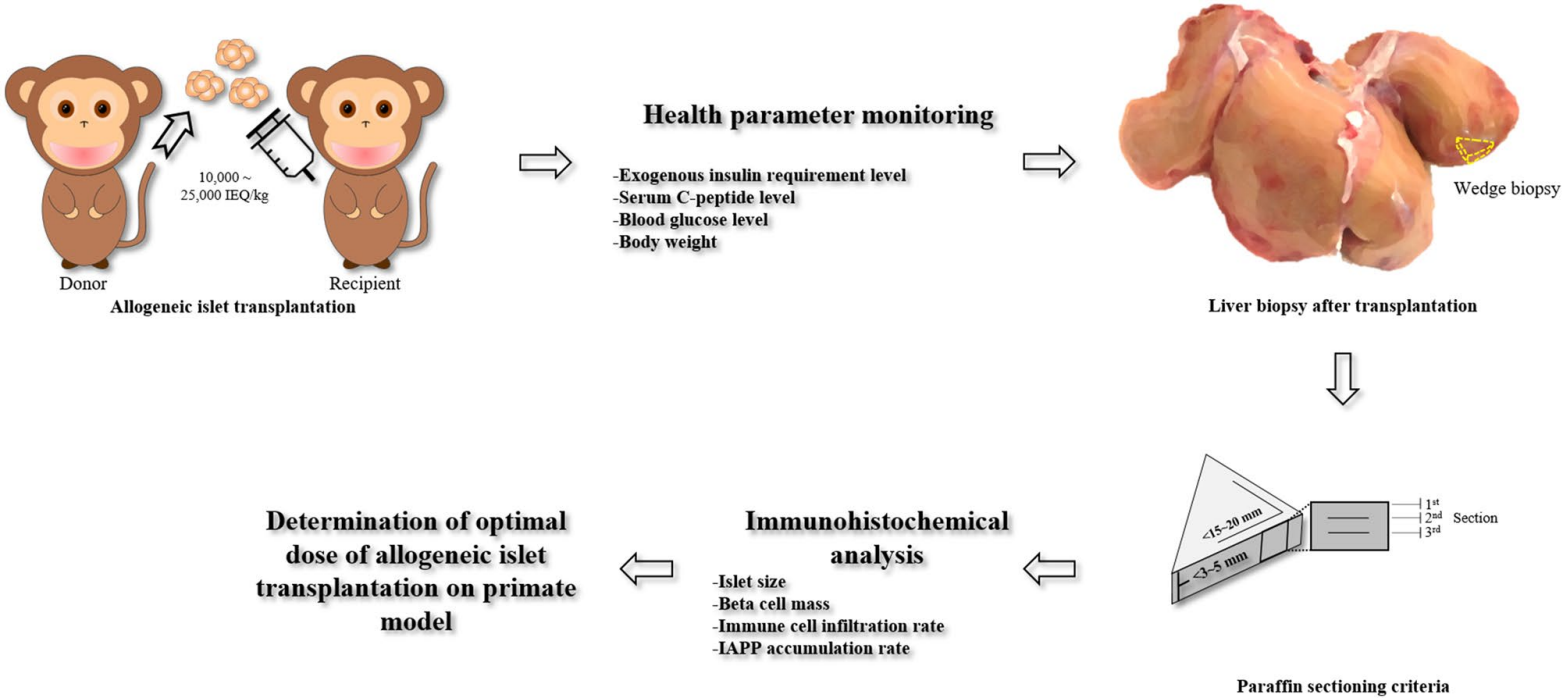
Scheme for allogeneic islet transplantation and liver biopsy process. Each diabetic recipient monkey received 10,000– > 25,000 IEQ/kg islets from a donor monkey. After transplantation, the health parameters of the recipient monkey were monitored, and a liver biopsy was conducted. The optimal dose of transplanted islets was determined after an analysis of the immunohistochemistry and insulin independence of each recipient monkey.

Eleven monkeys using an immunosuppressant regime successfully received allogeneic islets from eleven donor monkeys. Eight monkeys (A, B, C, D, H, I, J, and K) underwent rabbit anti-thymocyte globulin (ATG, Genzyme, Cambridge, MA) induction alone, and 3 monkeys (E, F, and G) in group 2-1 underwent combined anti-CD20 monoclonal antibody, rituximab (RTX, MabThera, Roche Pharma, Schweiz), and ATG induction (Supplementary Figure S1 and Table S1). The mean age and body weight at the time of transplantation were 50.5 (41–63) months and 3.7 (2.4–4.6) kg (Table 1). The mean dose of transplanted islets was 10,000 (group 1, N = 2 monkeys), 19,750 (19,500–20,000) (group 2-1, N = 2), 20,000 (group 2-2, N = 3) and 26,095 (25,000–27,000) (group 3, N = 4) IEQ/kg (Fig. 1 and Table 1). The characteristics of transplanted islets were not significantly different in terms of viability, insulin secretion, or purity (Supplementary Table S1). Before the start of the experiment, eleven islet recipient monkeys and pancreas donor monkeys were selected based on the results of the anti-donor autoantibody test (Supplementary Table S2).

**Table 1:** Transplantation information for the eleven experimental cynomolgus monkeys.

Group (IEQ/kg)	Monkey ID	N	Sex	Transplanted islet Dose (IEQ/kg)	Induction regimen	Age (TPL day, month)	Body weight (kg)	F/U (day)
1 (10,000)	A	2	M	10,000	ATG	41	4.0	89
	B			10,000		53	4.3	63
2-1 (20,000)	C	2	M	20,000	ATG	52	4.6	93
	D			19,500		63	3.6	98
2-2 (20,000)	E	3	M	20,000	ATG + RTX	48	4.2	47
	F			20,000		50	3.1	105
	G			20,000		45	2.4	140
3 (> 25,000)	H	4	M	25,000	ATG	49	3.2	195
	I			27,000		48	3.7	109
	J			26,882		46	3.3	160
	K			26,381		60	4.0	115

*IEQ* Islet equivalent number, *ATG* rabbit anti-thymocyte globulin, *RTX* rituximab, *TPL* Transplantation, *F/U* Follow-up day after TPL.

### Control of blood glucose levels depended on transplanted islet dose

**Figure 2:**
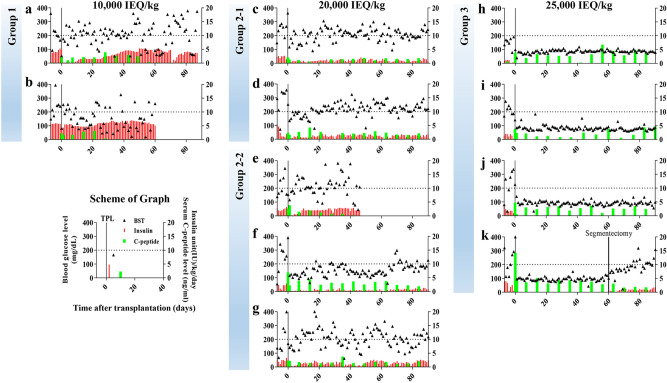
Blood glucose level control depended on the transplanted islet dose. After allogeneic islet transplantation, the blood glucose, C-peptide, and exogenous insulin administration levels of eleven monkeys were monitored for up to 90 days. (**a**,**b**) The group 1 monkeys received 10,000 IEQ/kg and showed uncontrolled blood glucose levels despite being given the most exogenous insulin. Monkeys A and B were terminated 87 and 60 days after transplantation, respectively, according to our experimental criteria. (**c**–**g**) The group 2-1 and 2-2 (induction as ATG + RTX) monkeys received 20,000 IEQ/kg and maintained blood glucose levels near 200 mg/dL through the provision of high levels of exogenous insulin. Monkey E expired unexpectedly but without significant changes in the relevant biological parameters. (**h**–**k**) The group 3 monkeys received > 25,000 IEQ/kg and achieved normal blood glucose levels with (**i**,**j**) or without (**h**,**k**) receiving exogenous insulin. The serum C-peptide level increased as the transplanted islet dose increased. (**k**) The blood glucose, C-peptide levels, and exogenous insulin requirements of Monkey K, who underwent a segment 2 segmentectomy 2 months after transplantation. Monkey K regressed to levels similar to those in group 2 and required daily exogenous insulin to maintain an appropriate blood glucose level.

**Figure 3:**
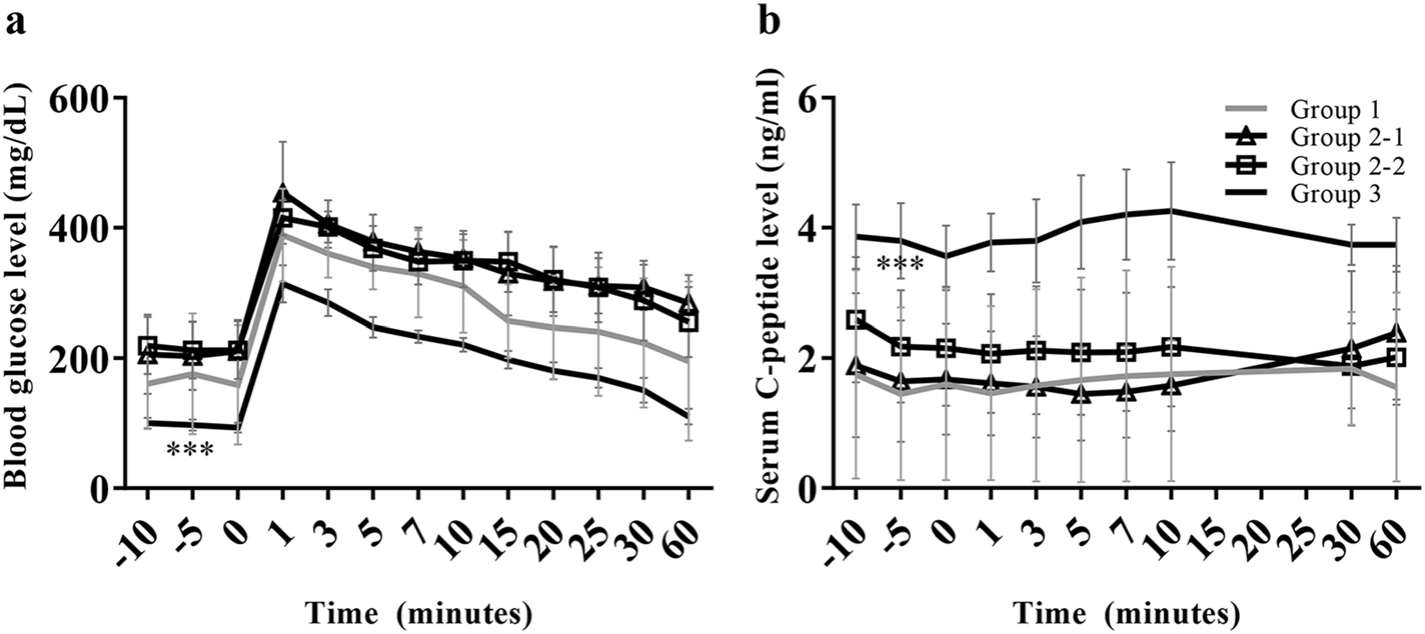
In the group that received a high dose of islets, the transplanted islets maintained their functioning. Intravenous glucose tolerance testing (IVGTT) was conducted one month after transplantation, and the (**a**) blood glucose level and (**b**) serum C-peptide level were analyzed. (**a**) The functioning of the islets in secreting insulin against an increased blood glucose level was the highest in group 3 (N = 4), but groups 1 (N = 2) and 2-1 (N = 2) and 2-2 (N = 3) did not differ from each other. (**b**) Serum C-peptide levels, which are related to insulin secretion, were also significantly higher in group 3 than in groups 1, 2-1 and 2-2. ***p < 0.001. The data are presented as mean ± SEM.

After transplantation, the mean follow-up duration for all the monkeys was 109.5 (47–195) days (Fig. 2). Two months after transplantation (between 30 and 60 days), monkeys A and B in group 1 had out-of-control fasting blood glucose levels despite daily exogenous insulin doses of 4.2 ± 0.1 and 6.3 ± 0.1 IU/kg/day, respectively. Their levels of serum C-peptide were 2.1 ± 9.5 (monkey A) and 0.1 ± 0.0 (monkey B) (Fig. 2a,b). Because of this, the experiment with monkey B was terminated 60 days after transplantation. Because monkey A continued to show high blood glucose levels despite exogenous insulin usage and had low serum C-peptide levels, as seen in monkey B, we terminated the experiment 87 days after transplantation (Fig. 2a). Between one and two months after transplantation (POD 30–60), the five monkeys in group 2-1 and 2-2 required 1.1 ± 0.1 (monkey C), 1.3 ± 0.1 (monkey D), 2.5 ± 0.1 (monkey E), 0.5 ± 0.0 (monkey F), and 1.2 ± 0.1 (monkey G) IU/kg/day of exogenous insulin to maintain of blood glucose levels near 200 mg/dL. In the same period, their levels of serum C-peptide were 1.4 ± 0.1 (monkey C), 2.4 ± 0.2 (monkey D), 1.4 ± 0.4 (monkey E), 3.0 ± 0.2 (monkey F), and 1.8 ± 0.2 (monkey G) ng/ml (Fig. 2c–g). 47 days after transplantation, monkey E expired unexpectedly and without significant changes in the relevant biological parameters. The group 3 monkeys required either no exogenous insulin (monkeys H and K) or a very low dose (monkey I used 0.2 IU/kg/day at 43 days after transplantation, and monkey J used 0.1 IU/kg/day at 36 and 46 days after transplantation) to maintain blood glucose levels below 100 mg/dL. Their levels of serum C-peptide were 3.3 ± 0.4 (monkey H), 2.2 ± 0.4 (monkey I), 2.5 ± 0.2 (monkey J), and 3.4 ± 0.3 (monkey K) ng/ml (Fig. 2h–k). Those results were maintained for three months (POD 90) after transplantation in each subject except monkey K. Interestingly, monkey K, who underwent segmentectomy (whole segment 2, left lateral lobe) at POD 60 because of unexpected bleeding during liver biopsy, showed dramatic changes in insulin independence, serum C-peptide level (decreased), and exogenous insulin needed to maintain glucose control after lobectomy (Fig. 2k). Intravenous glucose tolerance testing (IVGTT) 1 month after transplantation showed that the group 3 monkeys had significantly improved blood glucose levels (Fig. 3a) and serum C-peptide levels (Fig. 3b) than the monkeys in the other groups. Those results indicate that a dose of at least 25,000 IEQ/kg is required to achieve normal blood glucose levels without exogenous insulin after transplantation.

### Liver biopsy results after transplantation

One month after transplantation, group 1 was unavailable for biopsy because of health problems caused by uncontrolled blood glucose levels. In the same period, the group 2-1, 2-2 and 3 monkeys were biopsied, and 3, 5 and 5 liver tissue samples were obtained, respectively. Two months after transplantation, 3 liver tissue samples were obtained from monkey A. Monkey B was terminated in this period, in accordance with our criteria, and 49 liver tissue samples (whole segment 2, left lateral) were obtained. In the same period, 2, 6 and 9 liver tissue samples were biopsied from groups 2-1, 2-2 and 3, respectively. No islets were found in the tissues biopsied from group 1 two months after transplantation. One month after transplantation, 2, 9 and 17 islets were found in samples from groups 2-1, 2-2 and 3, respectively. Two months after transplantation, 5, 54 and 39 islets were found in samples from groups 2-1, 2-2 and 3, respectively. Information from the liver biopsies performed one and two months after transplantation is summarized in Table 2.

**Table 2:** Results of liver biopsies and detected islets.

Group (IEQ/kg)	Monkey ID	1 Month			2 Month		
		Biopsied tissue number	Biopsy site (segment)	Detected islet number (islets)	Biopsied tissue number	Biopsy site (segment)	Detected islet number (islets)
1 (10,000)	A	–	–	–	3	2	0
	B	–	–	–	49	2	0
2-1 (20,000)	C	2	3	2	1	1	0
	D	1	3	0	1	2	5
2-2 (20,000)	E	3	2	0	0	–	–
	F	1	1	6	5	2	25
	G	1	1	3	1	2	29
3 (> 25,000)	H	1	1	0	1	2	0
	I	1	2	0	2	2	2
	J	1	3	12	1	2	1
	K	2	1	5	5	2	36

### Characteristics of grafted islets

**Figure 4:**
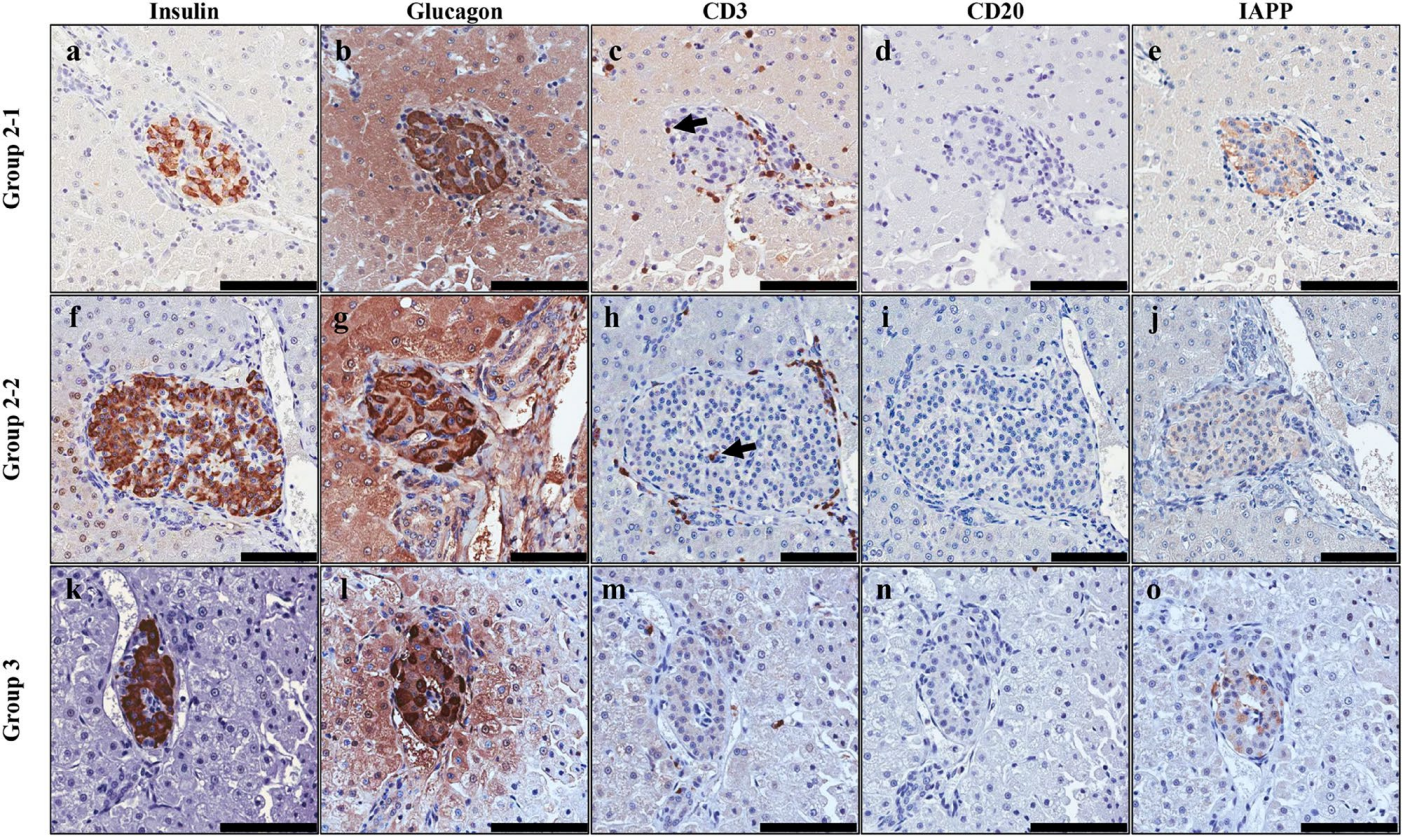
Representative results from the immunohistochemical analysis of islets found in liver biopsies conducted one month after transplantation. Liver biopsies were performed in group 2-1 (**a**–**e**), group 2-2 (**f**–**j**), and group 3 (**k**–**o**). In Group 1, no liver biopsies were performed due to the subjects’ poor health caused by high blood glucose levels. Insulin (**a,f**,**k**) and glucagon (**b**,**g**,**i**) were expressed in high amounts within the islets of groups 2-1, 2-2 and 3. Small numbers of CD3^+^ T cells (arrowed) (**c**,**h**,**m**) and CD20^+^ B cells (**d**,**i**,**n**) were detected in the islet areas of both groups 2-1, 2-2 and 3, but they were higher in both group 2-1 and 2-2. IAPP (**e**,**j**,**o**) was slightly expressed in all parts of the islets. Brown indicates the target protein in each result (scale bars = 100 μm).

**Figure 5:**
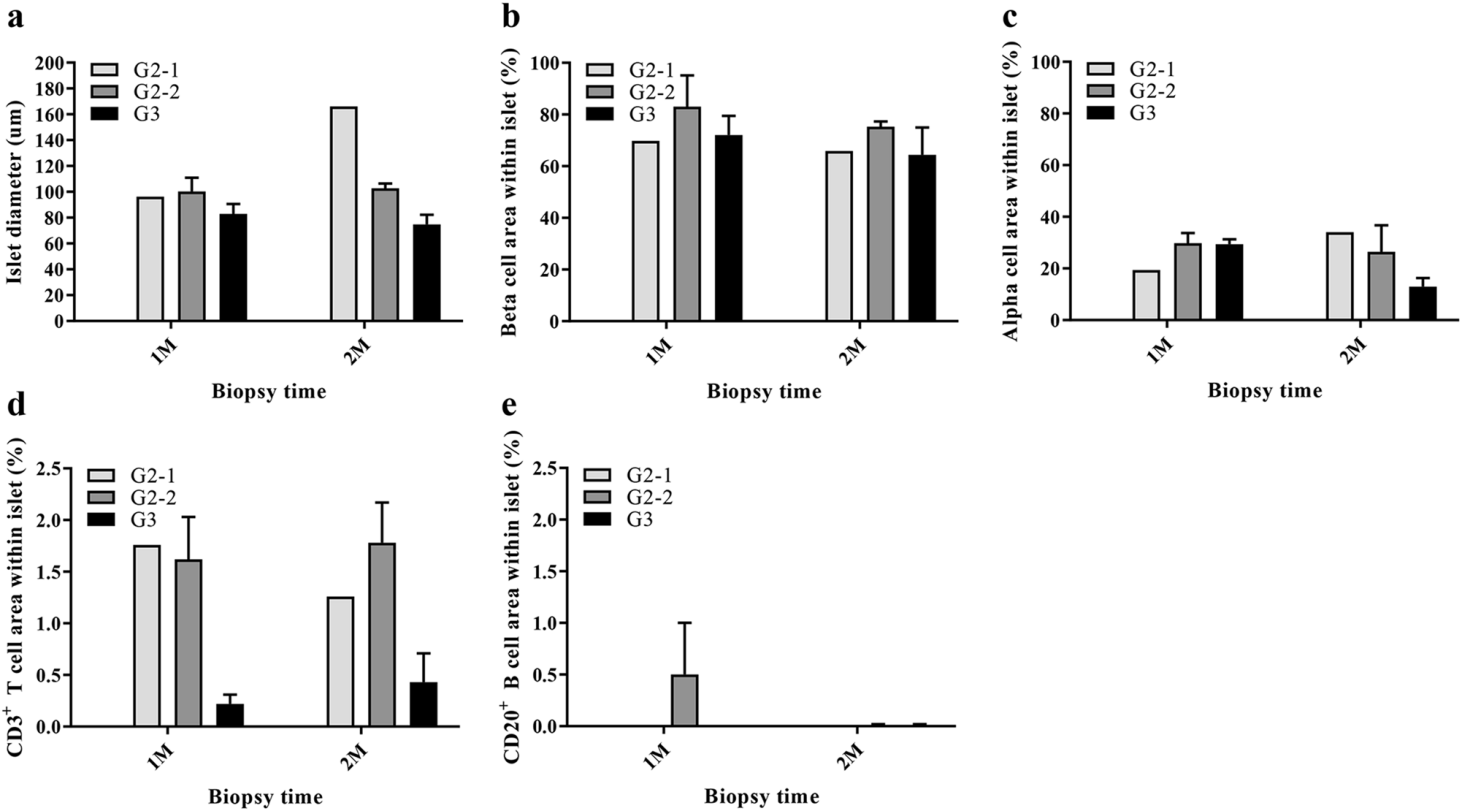
The characteristics of islets after transplantation. The characteristics of the islets found 1 month and 2 months after transplantation were analyzed using the Positive Pixel Count algorithm v9.1. (**a**) Islet size and (**b**) beta cell expression rate within the islet area was slightly higher in groups 2-1 and 2-2 than in group 3 both 1 and 2 months after transplantation. However, this difference was not statistically significant. (**c**) The alpha cell expression rate within the islet area did not differ statistically 1 month after transplantation, but it was significantly higher in group 2 after 2 months. (**d**) The CD3^+^ T cell infiltration rate within the islets was higher in group 2-1 and 2-2 in the entire period, but did not differ statistically. (**e**) The rate of infiltration of CD20^+^ B cells into the islets was only slightly higher in group 2-2 at 1 month. The data are presented as mean ± SEM.

The characteristics of the islets found in biopsied tissue were determined by quantifying their size (diameter, µm) and the expression rates (%) of beta cells, alpha cells, CD3^+^ T cells, CD20^+^ B cells, and IAPP within the islet area. Figure 4 shows representative results from various immunological stains of islets found in each group of liver tissues biopsied 1 month after transplantation (Fig. 4). The diameter of islets was greatest in group 2-1 at 2 months, but there was no significant difference among groups or periods (Fig. 5a). The beta cell area and alpha cell area also did not vary among groups or periods (Fig. 5b,c). Infiltrated CD3^+^ T cells within islets were remarkably increased in groups 2-1 and 2-2 compared with group 3 in all periods, but no statistically significant difference was found (Fig. 5d). CD20^+^ B cells showed a remarkable increase in group 2-2 at 1 month, but, similar to CD3^+^ T cell, this difference was not significant (Fig. 5e). These results indicate that although there are limitations regarding the analyzed number of islets, the dose of transplanted islets can affect islet characteristics, especially the CD3^+^ T cell infiltration rate early after transplantation.

### Dose of transplanted islets affected IAPP accumulation within the islets

**Figure 6:**
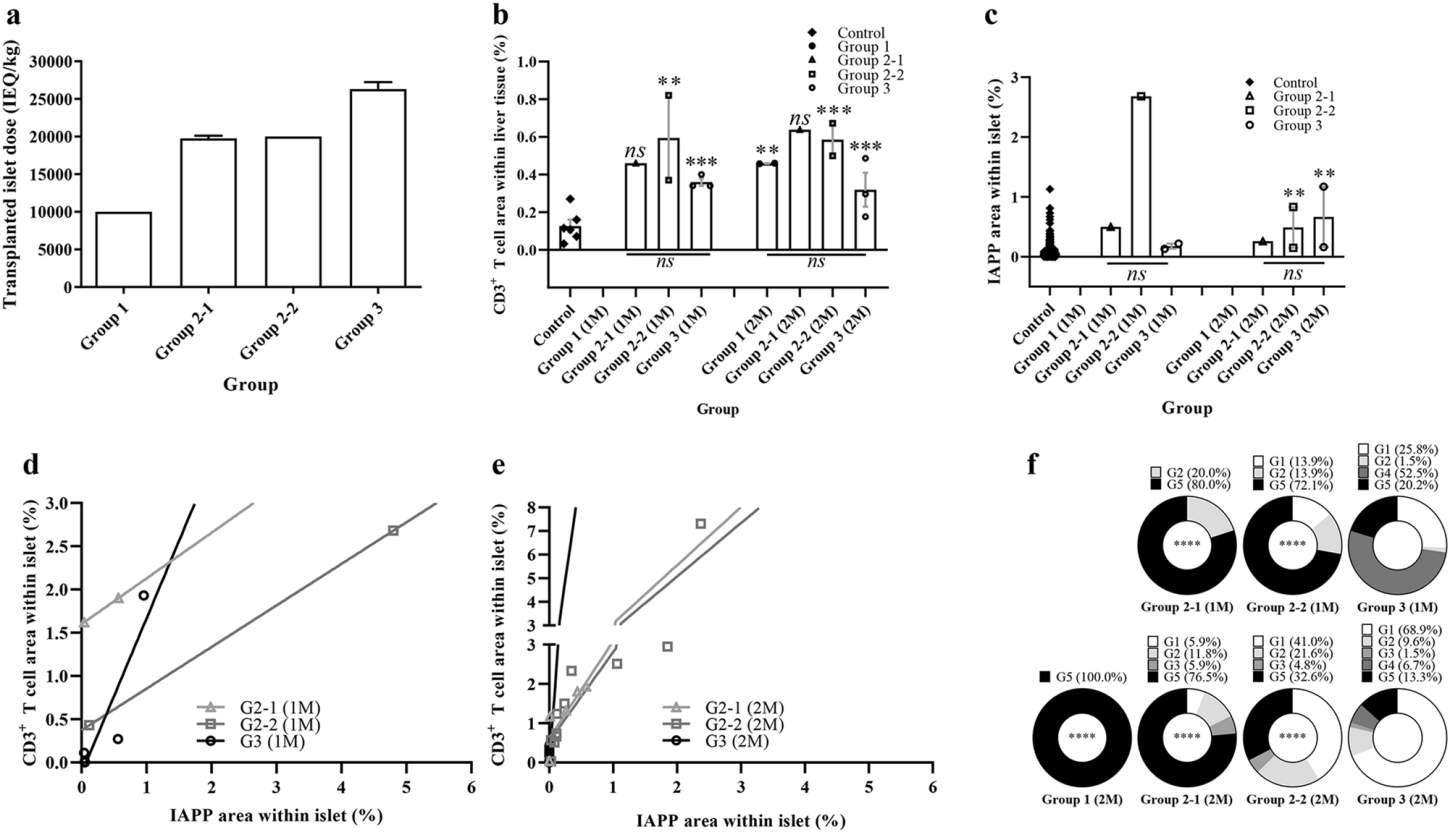
Dose of transplanted islets affected IAPP accumulation within the islets. Through immunohistochemical staining and an Aperio Positive Pixel Count algorithm (version 9.1) analysis, the IAPP expression rate within the islets was measured and graded according to the number of infiltrated T cells (grades 1–3) and the morphology of the islets (grades 4 and 5). (**a**) The mean number of transplanted islets was 10,000 (group 1, N = 2 monkeys), 19,750 (19,500–20,000) (group 2-1, N = 2), 20,000 (group 2-2, N = 3) and 26,095 (25,000–27,000) (group 3, N = 4). (**b**) All groups showed significantly increased CD3^+^ T cells in liver tissue compared with control liver (n = 6 tissues). Group 3 showed a lower CD3^+^ T cell expression level than other groups despite receiving the highest dose of islets. (**c**) The IAPP expression rate was significantly higher in group 2-2 (N = 1) one month after transplantation compare to normal pancreatic islet control (n = 431 islets). Statistically significant difference was not observed due to insufficient sample size. (**d**) The IAPP accumulation in islet cells and infiltration of CD3^+^ T cells was positively correlated at one month (group 2-1 [R^2^ = 1.0000, p = ns, n = 2 islets], group 2-2 [R^2^ = 1.0000, p = ns, n = 2] and group 3 [R^2^ = 0.8127, p = *, n = 5]). (**e**) The results were similar two months after transplantation (group 2-1 [R^2^ = 0.7151, p = ns, n = 5], group 2-2 [R^2^ = 0.8299, p = ****, n = 11]) and group 3 (R^2^ = 0.5329, p = **, n = 12). (**f**) The distribution of islets was classified by CD3^+^ T cell infiltration rate into islets. As the dose of transplanted islets increased, the distribution of normal functioning islets increased, while the ratio of grades 4 and 5, which rarely functioned, tended to increase as the dose of transplanted islets decreased. The statistical difference of the graph indicates the difference of each group to group 3. ns: p > 0.05, *p < 0.05, **p < 0.01, ***p < 0.001, ****p < 0.0001. The data are presented as mean ± SEM.

Next, we compared the transplanted islet dose, IAPP accumulation rate, and immune cell infiltration rate within the islets. The mean dose of transplanted islets was 10,000 (group 1, N = 2), 19,750 (19,500–20,000) (group 2-1, N = 2), 20,000 (group 2-2, N = 3) and 26,095 (25,000–27,000) (group 3, N = 4) IEQ/kg (Fig. 6a). The expression level of CD3^+^ T cells in islet-transplanted monkey liver tissue stained with CD3 was 0.46 ± 0 (group 2-1, N = 1), 1.03 ± 0.66 (group 2-2, N = 2) and 0.36 ± 0.02 (group 3, N = 3) one month after transplantation, and 0.46 ± 0.00 (group 1, N = 2), 0.64 ± 0 (group 2-1, N = 1), 0.59 ± 0.09 (group 2-2, N = 2) and 0.32 ± 0.09 (group 3, N = 2) two months after transplantation, respectively. The expression level of CD3^+^ T cells in normal liver tissue was 0.13 ± 0.03 (N = 6). CD3^+^ T cell area within liver area was significantly increased in islet-transplanted monkey liver compared to normal liver, but there was no significant difference among groups one month after transplantation. Two months after transplantation, there was no difference among groups 1, 2-1 and 2-2; however, group 3 exhibited significantly lower values than other groups (Fig. 5b). IAPP expression rate within the islet area was significantly higher in all groups and periods compared with normal pancreatic islets (0.05 ± 0.03, n = 431 islets) (Fig. 6c). The accumulation of IAPP within islets was relatively higher in the group receiving 20,000 IEQ/kg 1 month after transplantation compared with the group that received 30,000 IEQ/kg. However, no significant difference was found among groups or periods (Fig. 6c).

Next, to investigate the correlation between accumulated IAPP level within islets and the infiltration of CD3^+^ T cells into islets, the complete data set 1 month and 2 months after transplantation was analyzed (Supplementary Table S3). Interestingly, the results of linear regression analysis of islets 1 month after transplantation showed a positive correlation between IAPP accumulation in islet cells and infiltration of CD3^+^ T cells (group 2-1 [R^2^ = 1.0000, n = 2], group 2-2 [R^2^ = 1.0000, n = 2] and group 3 [R^2^ = 0.8127, p = 0.0366, n = 5]) (Fig. 6d). The results were similar two months after transplantation (group 2-1 [R^2^ = 0.7151, p = 0.0711, n = 5]), group 2-2 [R^2^ = 0.8299, p < 0.0001, n = 11]) and group 3 [R^2^ = 0.5329, p = 0.0070, n = 12]) (Fig. 6e).

To investigate the distribution of infiltrated CD3^+^ T cells, we quantified CD3^+^ T cells within the islets and the environment surrounding biopsied islets using five grades (G) (Supplementary Figure S2). The grades of the islets in group 2-1 (N = 1) one month after transplantation were G1 (0%), G2 (20.0%), G3 (0%), G4 (0%), and G5 (80.0%). The grades of the islets in group 2-2 (N = 2) one month after transplantation were G1 (13.9 ± 1.4%), G2 (13.9 ± 1.4%), G3 (0%), G4 (0%), and G5 (72.1 ± 2.9%). The grades of the islets in group 3 (N = 2) one month after transplantation were G1 (25.8 ± 7.6%), G2 (1.5 ± 1.5%), G3 (0.0%), G4 (52.5 ± 8.1%), and G5 (20.2 ± 2.0%). In group 1 (N = 1), two months after transplantation, only G5 islets were found. The grades of the islets in group 2-1 (N = 1) two months after transplantation were G1 (5.9%), G2 (11.8%), G3 (5.9%), G4 (0.0%), and G5 (76.5%). The grades of the islets in group 2-2 (N = 2) two months after transplantation were G1 (41.0 ± 29.6%), G2 (21.6 ± 1.0%), G3 (4.8 ± 1.1%), G4 (0.0%), and G5 (32.6 ± 29.7), and the islets in group 3 (N = 3) were G1 (68.9 ± 17.4%), G2 (9.6 ± 9.6%), G3 (1.5 ± 1.5%), G4 (6.7 ± 6.7%), and G5 (13.3 ± 10.2%) (Fig. 6f). As the dose of transplanted islets increased, the distribution of normal functioning islets increased, while the ratio of grade 4 and 5 islets, which rarely functioned, tended to increase as the dose of transplanted islets decreased (Fig. 6f). These results indicate that IAPP expression level increases as the number of transplanted islets decreases, and that IAPP is highly accumulated early after transplantation. These factors may affect the infiltration of T cells into islets.

## Discussion

Our present study demonstrates that > 25,000 IEQ/kg is the islet dose sufficient to achieve an exogenous insulin–independent normal blood glucose level after allogeneic islet transplantation when using a recent clinical immunosuppression protocol. The group that received 20,000 IEQ/kg required exogenous insulin to maintain blood glucose levels close to the reference value. This result is consistent with previous studies. The group that received 10,000 IEQ/kg had to be terminated from the experiment 2 months after transplantation to meet our criteria because of their high level of exogenous insulin demand after transplantation and unregulated blood glucose levels despite supplemental insulin. Upon termination, we found no functional islets in the biopsied liver. According to our previous report, segment 2 accounts for about 20% of the islets transplanted to the entire liver^32^. Two months after transplantation, one monkey (K) in group 3, which received > 25,000 IEQ/kg, showed indicators of diabetes (blood glucose level, serum C-peptide level, and insulin requirement) and regressed similarly to the monkeys in group 2 after we resected the whole of segment 2. That suggests that > 25,000 IEQ/kg is an optimal dose for achieving a normal blood glucose level after transplantation without exogenous insulin. However, that result is from only one monkey, so more detailed dose studies between 20,000 and > 25,000 IEQ/kg are needed.

ATG and rituximab are the immunosuppressive therapies used for T and B cell-regulation in clinical transplantation settings, and they are also used in allogeneic islet transplantation in NHPs^14,16–19,26^. In our three monkeys that used both ATG and rituximab, there were no differences in the factors related to the normalization of blood glucose level, especially the B cell infiltration affected by rituximab. This result is presumed to have been affected by our method of inducing diabetes, in which we removed the pancreas and spleen together, as previously described^18,33^.

The characteristics of the islets found in the liver biopsy after transplantation differed slightly in each group, but the size, insulin expression level, and glucagon expression level differences were not significant^32,34^. On the other hand, the IAPP expression level within the islets and CD3^+^ T cell infiltration seemed to correlate with each other. In diabetic monkeys, it is difficult to explain clearly why doses above 25,000 IEQ/kg were able to achieve normalization of blood glucose levels after transplantation without exogenous insulin. However, the expression level of IAPP within the islets correlates with the infiltration rate of CD3^+^ T cells doses between the 20,000 IEQ/kg and > 25,000 IEQ/kg groups. We thus expect that the mechanism of IAPP production occurs alongside the insulin production process in beta cells, allowing higher IAPP levels to accumulate in the group that received a lower dose of islet cells under the same diabetic condition. When an insufficient number of islets is transplanted into a diabetic monkey, each cell must produce a larger amount of insulin to normalize the blood glucose level, which would allow a larger amount of IAPP to accumulate within the islets compared with the islets of monkeys that received a sufficient dose of transplanted islets. This expectation is consistent with our results 1 month after transplantation, in which diabetic monkeys that received a smaller number of islets showed higher islet levels of IAPP accumulation than those that received more islets (Fig. 6c).

The expression of T cells by the liver and transplanted islets after allogeneic islet transplantation well-known to be due to allogeneic islet transplantation itself. The difference in the amount of CD3^+^ T cells expressed in the liver of monkeys undergoing allogeneic islet transplantation compared to the liver of normal monkeys without islet transplantation supports this (Fig. 6b). However, it is interesting that, while the number of allogeneic islets exhibiting immunogenicity during transplantation is the highest in group 3, the amount of CD3^+^ T cells expressed in liver tissue after transplantation is lower than other groups. Due to the small number of samples, this could not be confirmed statistically, but group 3 had the highest number of transplanted islets and the lowest CD3^+^ T cell expression (Fig. 6b).

On the other hand, several studies have reported that IAPP activates T cells^28–31^. Based on those results, we predicted that the increased IAPP accumulation found in the group that received an insufficient dose of islets would attract T cells into the transplanted islets. Our results are consistent with that expectation. 20,000 IEQ/kg groups, which received an insufficient dose of islets, showed a higher level of T cell infiltration than group 3 (> 25,000 IEQ/kg) both 1 and 2 months after transplantation (Fig. 5d). However, in group 1, which received the smallest number of islets (10,000 IEQ/kg), no islets were found two months after transplantation (Fig. 5). Interestingly, the phenomenon of T cell infiltration of islets showed that as IAPP expression increased, the population of islets that did not function also increased, which seemed to accelerate T cell infiltration into the islets (Fig. 6f). This correlation was evident one month after transplantation, but not at two months (Fig. 6c,f). However, the distribution of islets seen at 1 month remained similar 2 months after transplantation. This suggests that IAPP accumulation occurs vigorously early after transplantation, and this processes continues similarly thereafter. Thus, the hyperglycemic environment can accelerate the accumulation of IAPP into transplanted islets, especially early after transplantation, suggesting that IAPP accumulation within islets will negatively affect post-transplant outcomes^26^. However, under our experimental design, the exact origin of T cells infiltrating islets could not be identified. Subsequent experiments with additional hematological and histological analysis are needed to clarify the origin of the T cells infiltrating islets.

One of the major limitations of the present study is the small number of biopsied islets and related histological information. The probability of finding islets in liver tissue obtained through a single edge biopsy is low. The excision of large amounts of the liver to ensure successful detection of islets would affect the health of the monkeys and could thus negatively affect the stability of both the blood glucose level and the study as a whole. Therefore, the number of islets that could be found in the biopsies was small, making statistically reliable results difficult to obtain. In our previous study, which we conducted around the same time as this study, the optimal liver biopsy site for successful islet detection was segment 1. Most of the biopsies in this study were performed in segment 2, which had less than half the density of islets per liver area found in segment 1^32^. To overcome that limitation, future studies should conduct biopsies in a site with a high success rate.

In conclusion, the optimal transplantation dose to achieve a normal blood glucose level without the need for exogenous insulin in an insulin-dependent diabetic cynomolgus monkey model was > 25,000 IEQ/kg. IAPP accumulation into islets increases as the dose of transplanted islets decreases. This phenomenon is particularly noticeable early after transplantation, and appears to promote the invasion of T cells into islets.

## Materials and methods

### Animals and animal care

Eleven cynomolgus monkeys (*Macaca fascicularis*) were supplied by ORIENT BIO Co. Ltd (Seongnam, Korea). The monkeys were isolated in individual cages. Water was given ad libitum, and biscuits were supplied twice a day (Certified Primate Diet 5048, LABDIET, St. Louis, MO, USA). Fresh fruits, vegetables, and nuts were also provided twice a day. The inhabited environment was maintained at a temperature of 25 ± 2 °C, 40–60% humidity, 1–5 mmHg positive pressure air conditioning, and 300 lx illuminance alternating with darkness every 12 h. All the procedures related to infection screening, housing, handling, care, and treatment in this study were performed as previously described^33^. This study was approved by the Institutional Animal Care and Use Committee of Orient Bio Laboratories (ORIENT–IACUC-14195), and the experiments were performed in accordance with the relevant guidelines and regulations. All methods were carried out in compliance with the ARRIVE guidelines.

### Donor-recipient monkey selection

Anti-donor autoantibody for selection of transplantation pairs was analyzed via flow cytometry^35–40^. Briefly, isolated donor PBMCs were incubated with recipient sera for 30 min at 4 °C than washed with FACS buffer (BD, San Jose, CA, USA). FITC Conjugated-Monkey IgG (Gamma Chain) Antibody (OARA04820) (AVIVA SYSTEMS BIOLOGY, San Diego, CA, USA) was then added. After incubating for 30 min at 4 °C, they were washed twice. PBMCs were further incubated with APC-H7 Mouse Anti-Human CD20 (BD PHARMINGEN, San Diego, CA, USA), which has cross-reactivity with cynomolgus monkey, for 30 min at 4 °C. After washing, PBMCs were fixed with 2% paraformaldehyde. Fixed cells were acquired and analyzed with LSR Fortessa (BD BIOSCIENCES, San Jose, CA, USA) with FlowJo software (treestar). Donor self-serum was used as the negative control, and pooled sera (N = 10) were used as the positive control. Mean fluorescence intensity (MFI) ratio was calculated as: MFI value of recipient sera / MFI value of the negative control. Donor-to-recipient mates were determined based on an MFI ratio of < 2.0.

### Induction and management of diabetes mellitus

Pancreatectomy and the induction, confirmation, and maintenance of insulin-dependent diabetes mellitus in cynomolgus monkeys were performed as previously described^33^. Briefly, the donor monkey’s pancreas was removed through subtotal (> 70% of the pancreas) or total pancreatectomy. The removed pancreas was used for islet isolation, and the donor monkey whose pancreas was removed became a recipient monkey after an injection of 60–80 mg/kg of streptozotocin (SIGMA, St Louis, MO, USA). Diabetes mellitus was diagnosed when the following criteria were satisfied: (1) sustained hyperglycemia (blood glucose level > 250 mg/dl), (2) fasting C-peptide level < 0.5 ng/ml, and (3) decrease in stimulated C-peptide response in the IVGTT. After the onset of diabetes and islet transplantation, the blood glucose level was monitored 2–4 times daily, and exogenous insulin (glargine: Lantus; SANOFI-AVENTIS, Bridgewater, NJ, USA, and glulisine: Apidra, SANOFI-AVENTIS) were used to maintain blood glucose levels < 200 mg/kg to protect the animals from hyperglycemia (Fig. 2).

### Isolation and culture of islets

Resected partial or complete pancreases from the donor monkeys were processed as previously described to isolate the islets^33^. Briefly, the islets were isolated from the pancreas using the modified Ricordi method with collagenase MTF C/T (ROCHE, Indianapolis, IN). The discontinuous Ficoll density gradient method was used to purify the islets. The purified islets were seeded with 30,000 IEQ per 150Ø Petri dish, and cultured with CMRL-1066 supplemented medium (CORNING, NY, USA) supplied with 10% fetal bovine serum (GIBCO-THERMO FISHER SCIENTIFIC, Waltham, MA, USA) and 1% antibiotics (GIBCO) in a humidified 5% CO_2_ atmosphere at 37 °C. Cultured islets were used the next day (mean culture time was 20 (18–22) hours). On transplantation day, cultured islets were washed with fresh culture media, then counted for calculation of transplanted dose. Counted islets were suspended in CMRL 1066 transplantation medium (CORNING, NY, USA) without fetal bovine serum.

### Purity, viability and functionality of isolated islets

On transplantation day, the purity, viability and functionality was evaluated as described^33^. After dithizone (DTZ, SIGMA, St. Louis, MO) staining of the cultured cell mixture on day 1, DTZ positive endocrine cells (islets) were counted and the islet equivalent number (IEQ) calculated, then total cells (DTZ negative + positive) counted. The purity of the cultured islets was determined as percentage of DTZ positive IEQ of total IEQ. To assess islet viability, 0.67 μmol/L of acridine orange (SIGMA) and 75 μmol/L of propidium iodide (SIGMA) were used to identify living and dead islets. Stained islets were observed on 488- (live) and 594-nm (dead) emission through a florescent microscope. Functionality was assessed by glucose-stimulated insulin secretion (GSIS) assay. First, 5 to 10 hand-picked islets (approximately 150 um in diameter) were incubated on a 24-well plate for 1 h in Krebs–Ringer bicarbonate buffer (KRBB) supplemented with 0.2% bovine serum albumin (SIGMA) under the same conditions as islet culture. After incubation, walls containing islets were washed twice with KRBB solution, then low glucose solution (2.8 mM glucose with KRBB solution) was added and incubated for 1 h. After incubation, insulin containing low glucose solution was collected for analysis. Then, walls were washed twice, high glucose solution added (28 mM glucose with KRBB solution) and incubated for 1 h. After incubation, the incubated high glucose solution was collected. The secreted insulin level of the collected low and high glucose KRBB solutions was measured using a human insulin ELISA kit (MERCODIA, Uppsala, Sweden) according to manufacturer's instructions. The stimulation index (SI) value was determined as the insulin concentration of the high glucose solution divided by the insulin concentration of the low glucose solution.

### Allogeneic islet transplantation and perioperative management

Initially, we planned for the follow-up time after transplantation to be 3 months (90 days). In fact, we stopped the experiment early for some monkeys in accordance with humanitarian treatment because the affected animals endured the following conditions for two continuous months after transplantation: (1) blood glucose level > 200 mg/dL, (2) exogenous insulin usage > 3 IU/kg/day, and (3) serum C-peptide levels < 0.5 ng/ml. The transplantation of cultured islets and perioperative management were performed as previously described^18,33^. Briefly, at least 30 days after confirmation of insulin-dependent diabetes mellitus, 11 monkeys underwent islet transplantation via the intraportal injection of isolated islets (Supplementary Table S2). According to the injected IEQ/kg, the monkeys were divided into three groups: group 1 (10,000 IEQ/kg, N = 2), group 2-1 and 2-2 (20,000 IEQ/kg, N = 2 and 3), and group 3 (> 25,000 IEQ/kg, N = 4) (Table 1). After inducing general anesthesia, we performed central venous access port insertion via the right internal jugular vein. Through this central line, all monkeys received ATG four times at 12-h intervals to a cumulative dose of 20 mg/kg as induction immunosuppression. RTX injections at a dose of 375 mg/m^2^ were added in three monkeys (E, F, G) in group 2-2 as combination induction immunosuppression. Laparotomy began with an upper midline incision. After a self-retractor was applied, the portal vein was isolated. Islet-mixed heparin (75 IU/kg) was infused through an 18-gauge angiocatheter inserted into the portal vein. After islet infusion was finished, the angiocatheter was removed, and the puncture site was closed with 6-0 Prolene sutures. After each monkey awakened from anesthesia, it was returned to its cage. The immunosuppression schedule for pre- and post-transplantation is summarized in Supplementary Figure S1.

### Postoperative management

Postoperative management were performed as previously described^18^. Briefly, the monkeys received oral FK506 (Tacrolimus, Prograf, ASTELLAS Pharma Europe Ltd, Addlestone, UK) and mycophenolate mofetil (MMF, Cellcept, ROCHE Pharmaceuticals AG, Basel, Switzerland) as maintenance immunosuppressive drugs. The trough levels of FK506 and MMF were measured at intervals of 2–3 days (Supplementary Figure S1). To prevent inflammatory events, etanercept was given on the day of the transplant (day 0) and on days 3 and 6. A subcutaneous injection of anakinra (Kineret, Swedish ORPHAN BIOVITRUM, Stockholm, Sweden) was also given daily from days 0 to 7 (Supplementary Figure S1). One month after transplantation, IVGTT was performed after 12 h of fasting. After sedation with ketamine, two blood samples were drawn for C-peptide and blood glucose measurements. Then, 0.5 g/kg of dextrose was given intravenously, and blood samples were drawn 1, 3, 5, 7, and 10 min thereafter. Blood samples were also drawn at 15, 20, 25, 30, and 60 min to measure the glucose disappearance rate. Serum C-peptide was measured using a radioimmunoassay kit developed for human plasma (C-Peptide IRMA kit; IMMUNOTHECH, Beckman Coulter Inc., Prague, Czech Republic), which shows 90% cross-reactivity with plasma from cynomolgus monkeys. The acute C-peptide response was calculated as the difference between the mean C-peptide after glucose infusion and C-peptide at baseline (Figs. 2 and 3).

### Liver biopsy and immunohistochemical staining

Liver biopsies and the immunohistochemical analyses were conducted following our previously published criteria^32^. Briefly, to observe and investigate the environment of the grafted islets after transplantation, a protocol liver biopsy was performed one month (30 days) and two months (60 days) after transplantation, dependent on each monkey’s health condition. Monkey K of group 3 received a left lateral lobectomy 2 months after islet transplantation to assess the continuation of insulin independence after the removal of a considerable volume of islets from the grafted liver. The monkeys underwent a laparotomy to expose the liver, and then segment 1 or 2 of the liver was excised. The biopsied liver tissues were fixed in 10% neutral buffered formalin for 24 h and then embedded in paraffin. Each paraffin block was sliced into 30 paraffin slides that were 4 μm in thickness in a total of three sections, as previously described^32^ to produce 10 slides/section (Fig. 1). The slides produced in each section were stained for target proteins in the following order: hematoxylin and eosin (1st slide), anti-insulin from abcam (ab6995) (2nd slide), anti-CD3 from DAKO (A0452) (3rd slide), anti-CD20 from DAKO (M0755) (4th slide), anti-amyloid oligomers from ABCAM (ab126892) (5th slide), and anti-glucagon from ABCAM (ab92517) (6th slide). After deparaffinization and heat retrieval of the epitope, each slide was stained with the target protein for the 1^st^ antibody. To visualize of each target protein as brown, 3,3′-diaminobenzidine tetrahydrochloride staining was performed using a DAKO EnVision system (DAKO**,** Santa Clara, CA, USA) according to the manufacturer’s instructions (Fig. 4). As a control for the expression rate of CD3^+^ T cells present in islet-transplanted liver, liver tissue was obtained from six normal monkeys not related to this study and stained with CD3 in the manner described above. As a control for IAPP accumulation in islets found on liver biopsy and insulin-positive areas within the islet area of normal islets, pancreas tissue was obtained from three normal monkeys not related to this study and stained with IAPP and insulin in the manner described above.

### Analysis of immunohistochemically stained images

The immunohistochemistry slide images were analyzed as previously described^32^. Briefly, an image file of each stained slide was acquired using a ScanScope AT slide scanner (LEICA BIOSYSTEMS, Wetzlar, Germany) and Aperio ScanScope software (LEICA BIOSYSTEMS). Using the Aperio Positive Pixel Count algorithm (version 9.1) in the Aperio ImageScope program (version 12.1.0.5029; LEICA BIOSYSTEMS), we obtained the islet diameter (μm), islet area (μm^2^), and insulin-, glucagon-, CD3-, CD20-, and IAPP-positive areas within the total islet area (%) (Fig. 5). Because of the non-uniform shape and size of the islets and the change in the position of the islets on the slides from a continuous paraffin section, the number of islets for which we could analyze all the characteristics was small. To compensate for that, we analyzed the statistical significance of the characteristics between islets containing all the characteristics (complete data set) and islets containing only some of the characteristics (incomplete date set). As a result, we confirmed that there were no statistically significance differences in the remaining characteristics except for the size of the islets of group 2-2 and 3 at 2 months. Therefore, we used the incomplete data set (Supplementary Table S3).

### CD3^+^ T cell infiltration rate grade criteria

The CD3^+^ T cell infiltration rate into the islets was graded using the following criteria: (1) Grade 1—CD3^+^ T cells infiltrated into < 1% of insulin-expressing islets, (2) Grade 2—CD3^+^ T cells infiltrated into 1–5% of the insulin-expressing islets, (3) Grade 3—CD3^+^ T cells infiltrated into > 5% of the insulin-expressing islets, (4) Grade 4—Massive infiltration of CD3^+^ T cells into islets rarely expressing insulin (< 1% insulin), (5) Grade 5—Massive infiltration of CD3^+^ T cells into islets not expressing insulin (Supplementary Figure S2).

### Statistical analysis

The IVGTT results were analyzed using one-way ANOVA with Bonferroni`s multiple comparison post hoc test in GraphPad Prism version 5.00 (GRAPHPAD SOFTWARE, San Diego CA, USA) (Fig. 3a,b). The characteristics of the biopsied islets and tissue were analyzed using unpaired t testing with GraphPad Prism version 5.00 (GRAPHPAD SOFTWARE) (Figs. 5, 6b, and Supplementary Table S3). The correlation between IAPP expression in islet cells and CD3^+^ T cell infiltration was analyzed by liner regression analysis (GRAPHPAD SOFTWARE) (Fig. 6d,e). The distribution of islets infiltrated with CD3^+^ T cells was analyzed using the chi-square test (GRAPHPAD SOFTWARE) (Fig. 6f). All data are presented as means ± standard error of the mean (SEM).

## Supplementary information

Supplementary Information.

## Data Availability

The datasets generated during and/or analyzed for the current study are available from the corresponding author upon reasonable request.
