# Supplementary material for: Optimal allogeneic islet dose for transplantation in insulin-dependent diabetic Macaca fascicularis monkeys
Source: Sci Rep. 2021 Apr 21;11:8617. doi: 10.1038/s41598-021-88166-y (PMC8060424; doi:10.1038/s41598-021-88166-y)
Supplement: Supplementary file 1 — Supplementary Information. [file 41598_2021_88166_MOESM1_ESM.pdf]

Supplementary information

Optimal allogeneic islet dose for transplantation in insulin-dependent diabetic *Macaca fascicularis* monkeys

Geun Soo Kim,[1,2,3] Chan Woo Cho,[4] Jong Hyun Lee,[8] Du Yeon Shin,[1,2,3] Han Sin Lee,[2,6] Kyo Won Lee,[5,7] Yeongbeen Kwon,[3] Jae Sung Kim,[3,8] Heung-Mo Yang, [7,8] Sung Joo Kim,[7,8] and Jae Berm Park,[1,2,3,5,7]*

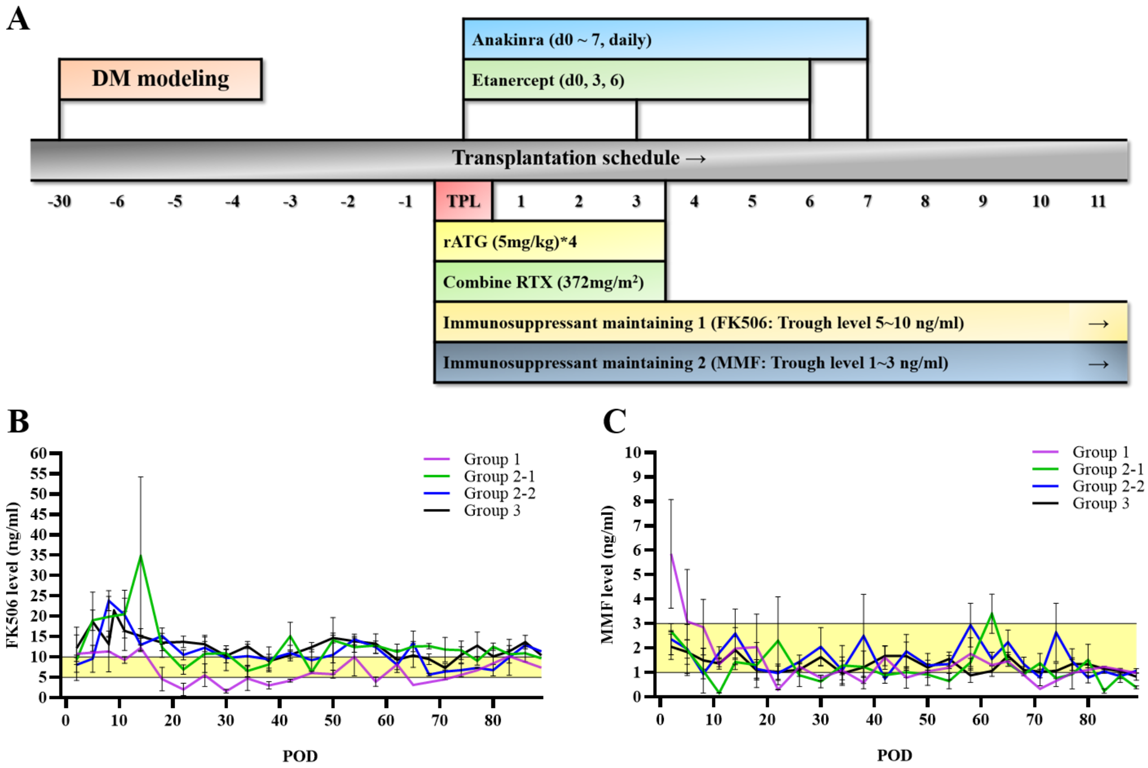

Supplementary Figure S1. The schedule of the immunosuppressant regime and trough level monitoring in monkeys. (**a**) Insulin-dependent diabetes mellitus was induced by pancreatectomy and streptozotocin (STZ) administration at least 30 days before transplantation. On transplantation day, the monkeys received rabbit anti-thymocyte globulin (ATG) four times at 12-hour intervals to a cumulative dose of 20 mg/kg as induction immunosuppression. Rituximab (RTX) injections at a dose of 375 mg/m2 were added as combination induction immunosuppression in monkeys E, F and G. All monkeys received oral tacrolimus (FK506) and mycophenolate mofetil (MMF) as maintenance immunosuppressive drugs. To prevent inflammatory events, etanercept was given on the day of transplantation (day 0) and on days 3 and 6. Subcutaneous injections of anakinra were also given daily from days 0 to 7. Trough levels of (b) FK506 and (c) MMF were measured at intervals of 2 to 3 days.

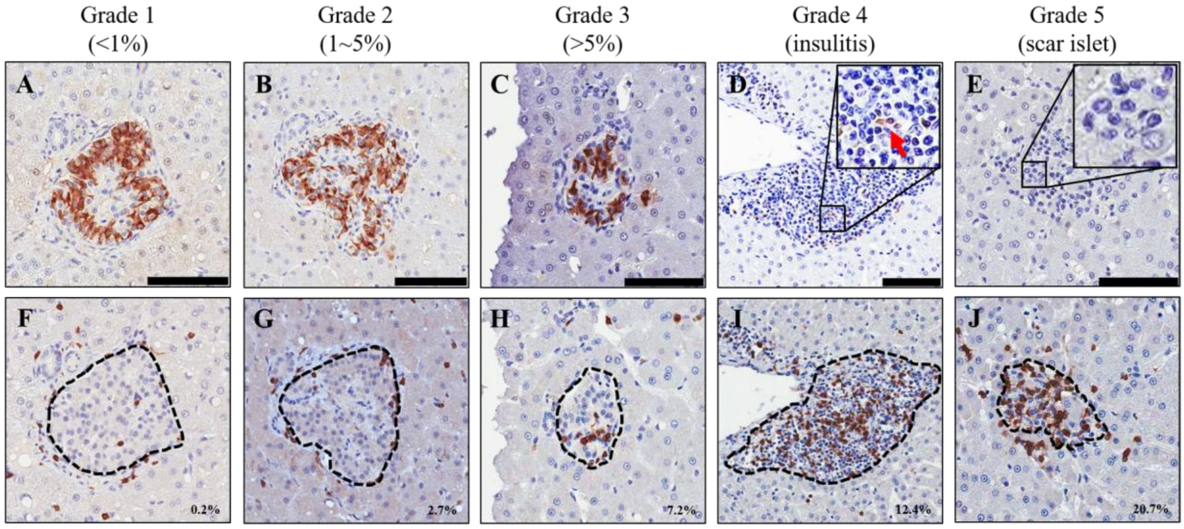

Supplementary Figure S2. Grading criteria for T cell–infiltrated islets. After immunohistochemistry staining, the detected islets were analyzed using the Aperio Positive Pixel Count algorithm (version 9.1). CD3+ T cell infiltration rate was graded according to the amount of infiltration within the islet area and morphology. (**a**), (**f**) Grade 1 was determined as a CD3+ T cell expression rate of less than <1%. (**b**), (**g**) Grade 2 was determined as a CD3+ T cell expression rate between 1 and 5%. (**c**), (**h**) Grade 3 was determined as a CD3+ T cell expression rate of more than >5%. (**d**) and (**i**) Grade 4, in which the islets rarely express insulin (red arrowed, <1%) because of near-destruction caused by a massive infiltration of T cells, is called *insulitis*. (**e**) and (**j**) Grade 5, in which the islets do not express insulin because of destruction by a massive infiltration of T cells, is called *scar islet*. Black dotted lines indicate islet boundaries. Brown indicates the target protein in each result (A–E = insulin, F–J = CD3). The CD3+ T cell expression rate is marked in the lower right of the second panel (scale bars = 100 μm).

Supplementary Table S1. Results of islet isolation and islet quality tests.

Characteristics		Group 1 (N = 2)	Group 2-1 (N = 2)	Group 2-2 (N = 3)	Group 3 (N = 3)	P value*
Donor age (months)		56.5±5.5	48.0±1.0	46.7±0.9	55.8±3.6	0.2400
Donor body weight (kg)		4.5±0.5	4.1±0.4	3.7±0.2	4.2±0.2	0.4124
Resected pancreas weight (g)		6.1±0.1	15.5±3.3	11.5±2.5	12.0±0.8	0.2416
Pancreatectomy type		Partial	Total	Total	Total	-
Cold ischemic time (min)		115±5.5	134±16.0	102±4.4	130±16.1	0.3464
Digestion time (min)		15.5±3.5	15.5±0.5	13.2±0.3	14.5±0.3	0.3274
Post purification	IEQ	72633.0 ±15300.0	96824.5 ±24758.5	124490.7 ±20309.5	137553.8 ±6873.7	-
	Isolation index	1.2±0.1	1.5±0.5	1.3±0.2	1.1±0.2	0.8895
Day 1 culture	IEQ	60600.5 ±9458.5	83529.5 ±11891.5	88627.3 ±1259.4	106879.8 ±6401.1	-
	Isolation index	0.84±0.1	1.3±0.4	1.2±0.2	1.1±0.2	0.3208
	Purity (%)	92.0±3.0	92.0±2.0	91.0±1.2	91.8±0.9	0.9544
	Viability (%)	94.5±2.5	96.0±1.0	94.7±2.8	94.0±2.5	0.9881
	Stimulation index	5.0±0.5	5.0±0.2	4.7±0.4	4.4±0.7	0.9024

*Kruskal-Wallis test.

Supplementary Table S2. Pancreas procurement and donor-recipient monkey selection results.

Group		G1		G2-1			G2-2			G3			P value*
Recipient	Monkey ID	**A**	**B**	**C**	**D**	**E**	**F**	**G**	**H**	**I**	**J**	**K**	
	(pancreatectomy type)	(P)	(P)	(P)	(P)	(T)	(T)	(T)	(T)	(T)	(T)	(T)	
Donor	Monkey ID	**C**	**D**	**E**	**F**	**H**	**I**	DM1	**J**	**K**	DM2	DM3	
	(pancreatectomy type)	(P)	(P)	(T)	(T)	(T)	(T)	(T)	(T)	(T)	(T)	(T)	
MFI ratio	IgG in donor	1.14	1.19	1.19	1.29	1.15	1.73	1.11	1.16	1.21	1.74	1.62	0.5147
	CD20 in donor	1.66	1.78	1.16	1.13	1.16	1.94	0.82	1.67	1.72	1.74	1.62	0.3840

DM, Donor monkey, P, Partial pancreatectomy, T, Total pancreatectomy, MFI, Mean fluorescence intensity, *Kruskal-Wallis test.

Supplementary Table S3. Results of the statistical analysis and numbers of islets found in liver biopsies.

Month	Group	Value	Diameter (um)		Beta cell expression rate (%)		Alpha cell expression rate (%)		$CD3^+$ T cell expression rate (%)		$CD20^+$ B cell expression rate (%)		IAPP expression rate (%)	
			IDS†	CDS††	IDS	CDS	IDS	CDS	IDS	CDS	IDS	CDS	IDS	CDS
1M	G2-1	Monkey N	1		1		1		1		1		1	
		Ave	96.18	96.18	69.77	69.77	19.36	19.36	1.76	1.76	0.00	0.00	0.50	0.50
		SEM	na†††		na		na		na		na		na	
		P value*	na		na		na		na		na		na	
	G2-2	Monkey N	2		2		2		2		2		1	
		Ave	100.32	123.27	83.06	83.82	29.79	34.66	1.62	1.79	0.50	0.00	2.68	2.68
		SEM	10.59	33.54	12.10	11.34	3.91	8.77	0.41	0.24	0.50	0.00	na	na
		P value	ns (0.1636)		ns (0.7273)		ns (0.2500)		ns (0.7518)		ns (0.9999)		ns (0.6650)	
	G3	Monkey N	2		2		2		2		2		2	
		Ave	82.92	106.34	72.05	81.20	29.36	23.59	0.22	0.55	0.00	0.00	0.17	0.15
		SEM	7.72	2.18	7.37	4.97	1.96	0.12	0.09	0.42	0.00	0.00	0.05	0.07
		P value	ns (0.1488)		ns (0.7503)		ns (0.7113)		ns (0.3497)		ns (0.9999)		ns (0.6371)	
2M	G2-1	Monkey N	1		1		1		1		1		1	
		Ave	166.01	166.01	65.87	65.87	34.05	34.05	1.26	1.26	0.00	0.00	0.26	0.26
		SEM	na		na		na		na		na		na	
		P value	na		na		na		na		na		na	
	G2-2	Monkey N	2		2		2		2		2		2	
		Ave	102.73	141.16	75.31	68.41	26.43	19.74	1.78	1.04	0.01	0.00	0.49	0.29
		SEM	3.76	7.20	2.01	1.38	10.31	7.00	0.39	0.81	0.01	0.00	0.34	0.29
		P value	** (0.0037)		ns (0.1045)		ns (0.1721)		ns (0.4147)		ns (0.5748)		ns (0.2459)	
	G3	Monkey N	3	2	3	2	3	2	3	2	3	2	2	2
		Ave	74.76	87.55	64.38	74.56	12.97	15.95	0.43	0.44	0.01	0.01	0.66	0.67
		SEM	7.50	6.90	10.58	5.26	3.35	3.31	0.28	0.44	0.01	0.01	0.51	0.51
		P value	* (0.0500)		ns (0.7622)		ns (0.7636)		ns (0.7762)		ns (0.9925)		ns (0.8065)	

*Unpaired t test, †IDS (incomplete data set), ††CDS (complete data set), †††na (not available, did not analyzed due to too small number to analysis or equal)
